# Recurrent Inhibition to the Medial Nucleus of the Trapezoid Body in the Mongolian Gerbil (*Meriones Unguiculatus*)

**DOI:** 10.1371/journal.pone.0160241

**Published:** 2016-08-04

**Authors:** Anna Dondzillo, John A. Thompson, Achim Klug

**Affiliations:** 1 Department of Physiology & Biophysics, University of Colorado, School of Medicine, Aurora, CO, 80045, United States of America; 2 Department of Neurosurgery, School of Medicine, University of Colorado, Aurora, CO, 80045, United States of America; Tokyo Medical and Dental University, JAPAN

## Abstract

Principal neurons in the medial nucleus of the trapezoid body (MNTB) receive strong and temporally precise excitatory input from globular bushy cells in the cochlear nucleus through the calyx of Held. The extremely large synaptic currents produced by the calyx have sometimes led to the view of the MNTB as a simple relay synapse which converts incoming excitation to outgoing inhibition. However, electrophysiological and anatomical studies have shown the additional presence of inhibitory glycinergic currents that are large enough to suppress action potentials in MNTB neurons at least in some cases. The source(s) of glycinergic inhibition to MNTB are not fully understood. One major extrinsic source of glycinergic inhibitory input to MNTB is the ventral nucleus of the trapezoid body. However, it has been suggested that MNTB neurons receive additional inhibitory inputs via intrinsic connections (collaterals of glycinergic projections of MNTB neurons). While several authors have postulated their presence, these collaterals have never been examined in detail. Here we test the hypothesis that collaterals of MNTB principal cells provide glycinergic inhibition to the MNTB. We injected dye into single principal neurons in the MNTB, traced their projections, and immunohistochemically identified their synapses. We found that collaterals terminate within the MNTB and provide an additional source of inhibition to other principal cells, creating an inhibitory microcircuit within the MNTB. Only about a quarter to a third of MNTB neurons receive such collateral inputs. This microcircuit could produce side band inhibition and enhance frequency tuning of MNTB neurons, consistent with physiological observations.

## Introduction

The medial nucleus of the trapezoid body (MNTB) is an auditory brainstem nucleus involved in the sound source localization pathway, as well as in a number of other auditory circuits[1–4]. It receives excitatory input from globular bushy cells (GBCs) located in the contralateral anterior ventral cochlear nucleus (aVCN) [5–10]. Large diameter axons of GBCs travel along the acoustic stria, cross the midline within the trapezoid body [10], and terminate on principal cells of the MNTB via a type of giant calyceal axo-somatic terminal termed the calyx of Held [5,11]. One single principal cell receives input from one GBC, but GBC axons occasionally branch within the MNTB to produce multiple calyces [5,10,12]. The MNTB is a major source of glycinergic inhibition to the ipsilateral medial and lateral superior olivary nuclei (MSO, LSO, respectively), the ventral and dorsal nuclei of the lateral lemniscus (VNLL, DNLL, respectively), and other targets [13–15]. Golgi staining and electron microscopy (EM) studies have characterized three types of neurons in the MNTB: stellate, elongate and principal cells ([5], cat) with the latter representing the majority (82%) of cells ([16], rat).

Due to the predominant glycinergic output of the MNTB, it has traditionally been considered a relay within the auditory pathway (reviewed in [17], but also see [18,19]). However, a number of anatomical and physiological reports suggest that MNTB cells also receive neural inhibition [1,9,20–24]. In particular, glycine and GABA positive label exists in non-calyceal presynaptic compartments terminating on the principal cell soma, as demonstrated by EM, as well as immunohistochemistry and light microscopy [25,26]. The GABA contribution to the inhibitory postsynaptic current decreases with age. Electrophysiological studies of *in-vitro* brainstem sections of the MNTB showed that the inhibitory glycinergic input produces large postsynaptic currents with very fast decays, and has the potential to shunt calyceal glutamatergic input [20,21,27]. Recordings from MNTB neurons *in-vivo* show several types of glycinergic effects acting on MNTB firing in different ways, including lateral inhibition. This lateral inhibition was blocked by applying the glycine receptor blocker strychnine [24], suggesting the presence of glycinergic inputs on MNTB principal neurons.

A recent anatomical study using bulk neuronal tracing in the mouse indicated the ventral nucleus of the trapezoid body (VNTB) as the major extrinsic source of glycinergic input to the MNTB [20]. Besides this extrinsic source, several studies have suggested an additional intrinsic source of inhibitory input to MNTB neurons. For example, tracing studies using neurobiotin or horseradish peroxidase (HRP) in the recording electrode observed that a minority of MNTB principal cells have axonal collaterals returning back to the vicinity of the originating cell (gerbil, [9] cat [23]; rat, [28]). While suggestive, these collaterals have never been investigated in more detail, and the question of whether they are actual synaptic inputs has never been verified.

In this work we test two main hypotheses. 1) Do collaterals of principal MNTB cell axons remain within the nucleus and 2) if so do they form inhibitory synapses on other MNTB principal cells?

## Materials and Methods

### Ethics Statement

All animal procedures were approved by the Institutional Animal Care and Use Committee (IACUC) of the University of Colorado Medical Campus (Permit number B-88412(05)1D. Furthermore, all applicable laws and regulations, as well as the PHS Policy were strictly followed. A total of 37 male and female gerbils, reared in our in-house colony at the UC Denver were used in these experiments. All animal procedures were approved by the University of Colorado School of Medicine Animal Care and Use Committee, and were conducted in accordance with National Institutes of Health standards on humane treatment of laboratory animals.

### *In-vitro* single cell filling and recording

*Slice Preparation*. Slices of brainstem were prepared from Mongolian gerbils (*Meriones unguiculatus*) of both sexes ranging in age from p13 to p17 (all stages after hearing onset). Animals were anesthetized by isoflurane inhalation (IsoFlo, Abbott Laboratories, USA) and decapitated. The brainstem was dissected out and cut into slices of 180 to 300 μm with a vibratome (VT1000S, Leica, Wetzlar, Germany) under ice-cold dissection ringer (125 mM NaCl, 2.5 mM KCl, 1 mM MgCl_2_, 0.1 mM CaCl_2_, 25 mM glucose, 1.25 mM NaH_2_PO_4_, 25 mM NaHCO_3_, 0.4 mM ascorbic acid, 3 mM myo-inositol, 2 mM pyruvic acid; all chemicals from Sigma–Aldrich, MO) bubbled for at least 15 min with 5% CO_2_-95% O_2_. Slices were transferred to an incubation chamber containing artificial cerebrospinal fluid (aCSF) (125 mM NaCl, 2.5 mM KCl, 1 mM MgCl_2_, 2 mM CaCl_2_, 25 mM glucose, 1.25 mM NaH_2_PO_4_, 25 mM NaHCO_3_, 0.4 mM ascorbic acid, 3 mM myo-inositol, 2 mM pyruvic acid; all chemicals from Sigma–Aldrich) and bubbled with 5% CO_2_-95% O_2_. Slices were incubated for up to 45 min at 37°C, after which the chamber was brought to room temperature. The dye fillings were performed within 4–5 hours of slicing.

### Single Cell dye loading

Single brain sections were placed in a recording chamber and viewed on a Zeiss Axioscope II microscope with a 40x water immersion objective, which was part of a standard patch clamp setup (described in [29]). In some experiments, neurons located in the MNTB were imaged and whole-cell patch clamped with glass electrodes. Patch pipettes (2.4–3.2 MOhm) were pulled from 1.5 mm borosilicate glass (Harvard Instruments, Kent, UK) using a DMZ Universal Puller (Zeitz Instruments, Munich, Germany) and filled with potassium gluconate internal solution (113 mM K-Gluconate, 4.5 mM MgCl_2_, 9 mM HEPES, 5 mM EGTA, 14 mM Tris2-Phosphocreatine, 4 mM Na_2_ATP, 0.3 mM tris-GTP, 1.5 mM CaCl_2_; pH adjusted to 7.25 with KOH; 295–300 mOsm; all chemicals from Sigma–Aldrich). After a whole-cell configuration was successfully obtained, the cell was left attached to the electrode for about 20 minutes without any further electrophysiological recordings to allow dye diffusion. Biocytin (Sigma-Aldrich, cat. B4261) at 3 mg/ml dissolved in internal pipette solution was used to label neurons.

In additional experiments, cells were labeled with an extracellular puff of dye solution delivered to the vicinity of the cell soma, followed by juxtacellular electroporation. For this method, similar glass pipettes as described above were used, but were filled with aCSF and 3 mg/ml biocytin. Extracellular puffs were delivered at 500 ms duration and a pressure ranging from 4–6 psi, delivered through a Picospritzer III (Parker Hannifin Corp, Cleveland, OH, USA) that was connected to the electrode. Electrical stimuli were 20 ms square pulses of 5–7 V repeated 20–50 times, delivered with an STG 2004 computer-controlled four-channel stimulator (Multi Channel Systems, Reutlingen, Germany) and a stimulation isolation unit (Iso-flex, AMPI, Jerusalem, Israel). To minimize the probability of unspecific label, the juxtacellular electroporation was obtained under visual control, whereby we identified the cell soma of the cell to be electroporated under the microscope with 40x objective. Then we brought the tip of the pipette onto the cell membrane, established a cell attached mode at 100–600 MOhms resistance similar to a “loose patch” configuration, and then applied the electroporation procedure. Due to the constant flow of 3ml/min of the recording bath through the chamber, the dye was washed from the bath at a steady speed, therefore additionally decreasing the probability of labeling nearby cells. The pipette was kept in place for about 2 minutes after the electroporation to allow dye diffusion.

### Sample sizes

We successfully filled 39 neurons in sections prepared from 37 gerbils aged P13 –P17 (summary in Table 1). 22 of these fillings neurons showed either only very short or no neurites labeled (total length of neurites less than 60 μm). In the remaining 17 neurons, the total length of neurites labeled in each neuron was at least 60 μm, not including the soma. In a few cases, labeled neurites were recovered without the originating soma, which may have become porous to the dye either during the patch clamp (and electrode removal) procedure, or the electroporation process.

**Table 1:** Total number of neurons labeled with biocytin juxtacellular electroporation, and patch clamp with dye diffusion in gerbil MNTB brain slices of different ages.

Age	P13	P14	P15	P16	P17	Total
**No. of neurons**	7	23	3	5	1	39

Most of the analysis was performed on coronal brain sections (35 cases). This plane was chosen because neurites largely remain within the section cut in this plane, meaning they only occasionally weave back and forth and if so, this is within a 30 μm range in the rostro-caudal plane relative to the soma. Importantly, neurites do not project in the rostro-caudal direction. If the latter was the case we should have seen punctate pattern formed by labeled neurites that exited the plane, which we have not observed. Furthermore, in additional experiments using sections cut in the horizontal and parasagittal plane, neurites typically traveled in the medio-lateral direction.

### Reconstruction of Labeled Cells

The following process was used to reconstruct the three-dimensional architecture of labeled cells. Mounted sections were imaged on an Olympus FV1000 confocal microscope (Olympus, Waltham, MA) using a 60x, 1.2 N.A., or a 20x, 0.75 N.A., or a 10x, 0.4 N.A. objective. One TRITC (547 nm) laser line was used for excitation of the dye that was used to fill the cell. For each filling, between 19 and 54 confocally acquired serial optical sections were recorded (mean = 40 sections per stack) and imported into Matlab 2014a with installed Image Processing Toolbox (Mathworks, Natick MA). To achieve the best visualization of the three-dimensional structure of the labeled cell, we sought to isolate pixels associated with fluorescently tagged dye label localized to the cell. In order to isolate these pixels, we derived an image mask of the cell’s outline that was applied to each section of the stack. The user-defined mask was drawn around the labeled components—leaving a ~20 pixel border—and not exclusively traced upon the visible structures. This method both excluded sources of noise (e.g., autofluorescence, and artifacts), as well as allowed semi-objective visualization of labeled cell architecture. Briefly, for each stack of images, the maximum projection (i.e., the maximum pixel intensity value for each [x, y] location in the image stack) was computed as a single matrix for the first channel of an RGB image. The pixel intensity values of the maximum projection matrix were normalized between 0 and 1 based on dividing each pixel intensity value by the maximum pixel value. The normalized maximum projection matrix was converted into a binary image in which pixel values > 0.75 = 1 and pixel values < 0.75 = 0. To further refine the cell outline, a pixel cluster detection algorithm (i.e., Matlab Image Processing Toolbox function ‘bwareaopen’) was applied to the matrix to detect all contiguous pixel clusters and exclude clusters < 40 pixels. At this stage, small areas of high pixel intensity artifact had been removed. Of the remaining contiguous pixel clusters in the binary image, the largest was associated with the outline of the labeled cell. To create the final cell outline image mask to be applied to each section, a user-defined polygon mask was traced around the largest contiguous pixel cluster in the maximum projection binary image. This image mask constructed of the user defined region-of-interest was applied to each individual optical sections of the imported image stack. Finally, pixel values extracted from each plane of the stack within the user-defined region-of-interest were used to generate a three-dimensional plot (Matlab function ‘patch’).

### Immunohistochemical Procedures

In each brain section, between 3 and 9 single neurons were targeted for labeling, depending on the size and accessibility of the MNTB in each section. After the cell fillings were completed, slices were recovered from the recording chamber and transferred to 4% paraformaldehyde (PFA) in phosphate buffered saline (PBS) for up to 2 hours. Sections then were either processed directly for immunohistochemistry, or re-sectioned. Slices selected for re-sectioning were transferred to 30% sucrose solution for up to 3 days, re-sliced into 50 μm thick sections on a freezing microtome (Leica SM 2010R, Buffalo Grove, IL) and subsequently processed for immunohistochemistry.

For immunohistochemical labeling, sections were incubated in 0.3% Triton X-100 (TX100; Sigma-Aldrich) and 5% normal goat serum (NGS; Jackson Immunoresearch Laboratories, West Grove, PA) in PBS for 60–120 minutes. Primary antibodies were diluted in 1% NGS and 0.3% TX100 in PBS, and the tissue was incubated for two days at 4°C. After primary antibody incubation, the tissue was rinsed several times with 2% NGS in PBS. Secondary antibodies were prepared in 1% NGS and 0.3% TX100, and sections were incubated at room temperature for 2 hours. After a final rinse, sections were mounted in Fluoromount-G (SouthernBiotech) medium. Mounted sections were imaged on an Olympus FV1000 confocal microscope using a 60x, 1.2 N.A., or a 20x, 0.75 N.A., or a 10x, 0.4 N.A. objective. Three laser lines were used for excitation of the dyes: 488nm, TRITC (547 nm), and 635 nm. Based on the axial dimensions of the voxels from 0.07 μm to 0.2 μm at the highest magnification scans of the immuno-positive clusters we reasoned the apposition of presynaptic and postsynaptic structures. For all identified terminals in which we observed positive immunohistochemical signal, we assessed apposition with the following criteria. Apposition required positive signals from both biocytin and immunohistochemical label to co-occur in at minimum two contiguous pixels.

#### Antibodies

The primary antibodies used in this study are listed in Table 2. The gephyrin mouse monoclonal antibody mAb7a (Synaptic Systems, cat. No: 147 011, RRID: AB_887717 Goettingen, Germany) was generated against an extract of rat spinal cord crude synaptic membranes [30,31] and shown in Western blots from extracts of rat brain membranes to bind specifically to a 93-kDa band of a membrane protein (gephyrin) [32,33]. Specificity of this antibody has also been shown previously in a gephyrin null mutant mouse, which resulted in no immunolabeling of gephyrin clusters [34].

**Table 2:** Summary of the primary antibodies used in this work. Final dilutions are based on the original stock concentrations, and were established experimentally.

Antigen	Description of Immunogen	Source, Host Species, Cat. #, Clone or Lot#, RRID	Dilution
gephyrin	Rat N-terminus of gephyrin	Synaptic Systems, mouse monoclonal antibody, cat. No: 147 011; Clone: mAb7a (GlyR7a)	1:2000
SNAP-25	Synthetic peptide corresponding to the N-terminus of human SNAP-25 (aa 9–29)	Sigma-Aldrich, rabbit IgG fraction of antiserum, cat. # S9684, Lot # 069K4784	1:10 000

The rabbit anti-SNAP-25 antibody Sigma-Aldrich (cat. No.: S9684, Lot No.: 069K4784, RRID: AB_261576) was isolated from the IgG fraction of antiserum against a synthetic peptide corresponding to the N-terminus of human SNAP-25 (amino acids 9–29 with C-terminally added lysine), which is identical to the sequence in rat, mouse and chicken. Specificity of this antibody has been shown by inhibition of immunoblotting after pre-incubation of the antibody with SNAP-25 immunizing peptide (amino acids 9–29 with a C-terminal lysine; manufacturer’s datasheet).

The following secondary antibodies were used (all from Invitrogen/Molecular Probes): goat anti-rabbit IgG conjugated with Alexa 647 (cat. No: A21245) and goat anti-mouse IgG conjugated with Alexa 488 (cat. No.: A11029). Both secondary antibodies were used at 1:1000 dilutions. For biocytin reaction an avidin conjugated with TRITC (ExtrAvidin-TRITC conjugate; Sigma-Aldrich cat. E3011) was used at 1:1000 dilution in the same solution as secondary antibodies.

## Results

The goal of our study was to test the hypothesis that axons from MNTB neurons form collateral inhibitory inputs onto neighboring MNTB neurons. We tested this hypothesis by labeling axons of single MNTB neurons, identifying potential recurrent collaterals, characterizing their terminals, and estimating the number of neurons that produce such collaterals. While previous studies have suggested their occurrence [9,23,28], these studies did not address how frequently these collaterals occur, or whether they actually produce synaptic terminals.

### Labeling of single neurons in the MNTB

**Fig 1:**
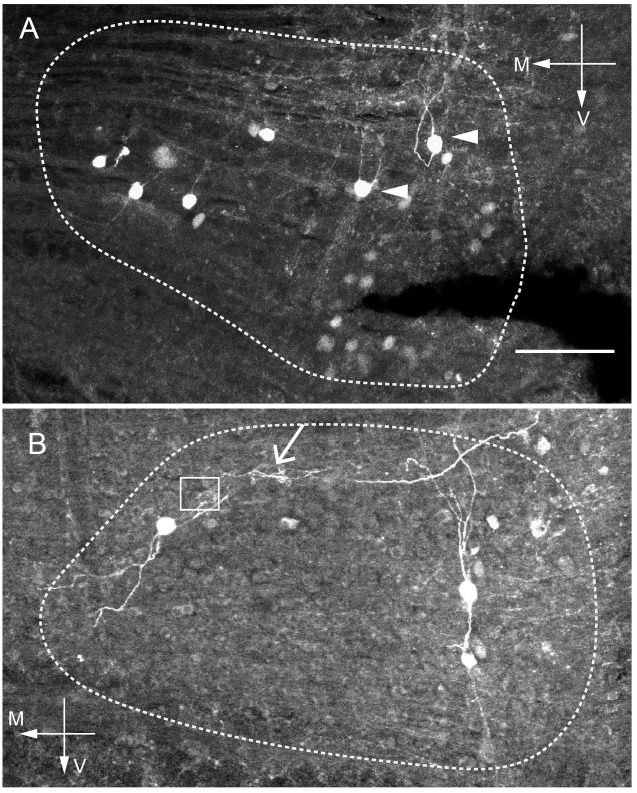
Single cell electroporation reveals putative MNTB to MNTB collaterals. A: brainstem section containing the MNTB with several biocytin-labeled neurons labeled with the single cell electroporation approach. The dashed line delineates borders of the MNTB based on the autofluorescence of the nucleus. Six neurons are labeled, two of them (arrowheads) have distinct neurites that turn within the MNTB. Note that dimmer cells, probably passively labeled by a puff do not have labeled neurites. B: Another example of brainstem section with MNTB and three labeled neurons located further apart, with one neurite travelling across the MNTB between the upper two cells. The red arrow indicates a location where neurites appear to intermingle. The red box indicates an area that is shown magnified in Fig 6A. Scale bar 100 μm in both panels.

**Fig 2:**
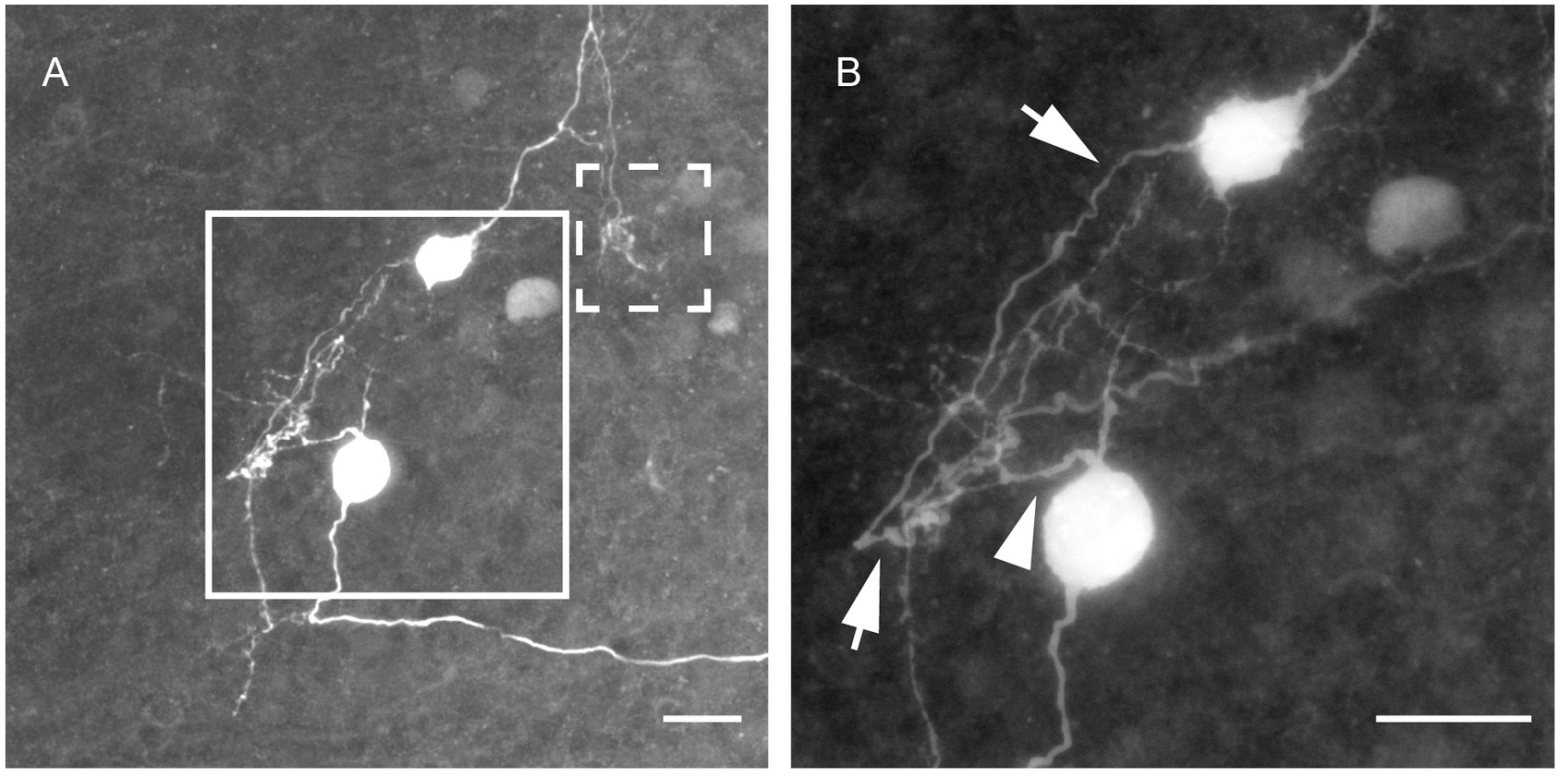
Tracing collaterals of MNTB principal cells requires sparse labeling of the neurons to avoid overlapping neurites. A: A neurite originating from the upper cell travels downward into close proximity of the lower neuron’s dendrites. Another neurite originating from the same (upper) cell travels upward and splits, to return and form a terminal in the dashed box. Maximal projection through a depth of 15.2 μm (19 virtual sections of 0.8 μm/slice = 15.2 μm). The dashed square is magnified in Fig 5D. B: Magnification of the area shown within the solid box in panel A, showing the neurite of the upper cell (arrows) spiraling near the area of the dendritic tree of the lower neuron (arrowhead). Average projection through a depth of 34.8 μm (58 virtual sections of 0.6 μm/slice = 34.8 μm). Scale bar in both panels: 20 μm.

We used both whole-cell, patch-clamp intracellular dye diffusion, as well as single cell electroporation approaches to label individual neurons in the MNTB. We decided to label only a small number of neurons per MNTB to reduce the likelihood of reconstructing too many labeled overlapping neurites, which would obfuscate origin of the neurites. Therefore, between 3 and 9 neurons per slice were labeled (Fig 1), but note that not every experiment resulted in a recoverable labeled neuron. Importantly, in neurons that might have passively taken up dye from the cell-adhered infusion, we never observed labeled neurites. Fig 1A shows 8 very bright cell somata, and multiple other ones that are lighter than background but dimmer than the eight bright cells. These dimmer cells are most likely an example of passive intake of the dye from the puff without cell attached electroporation which, never resulted in neurite labeling. Out of six labeled neurons located at least 2 cell diameters apart from each other (Fig 1A), two have very distinct neurites leaving the soma toward the dorsal aspect of the MNTB. Fig 1B shows the somata of three labeled neurons, plus one long neurite from an unidentified cell body spanning the medio-lateral length of the MNTB. The longer neurites tend to intermingle and form spirals apparently around each other, making it very challenging to trace their paths, (arrow in Fig 1B, see also Fig 2). The box in Fig 1B indicates the presence of a separate axonal formation that originates from an unidentified cell soma and is located at a different depth than the cell bodies pictured here.

The mosaic scan shown in Fig 2A is a maximal projection through the total depth of 15 μm of a P13 brainstem section of the MNTB. The two somata are located more than 40 μm away from each other. The putative axon originating from the upper cell travels downwards into the vicinity of the second soma. The magnified image in Fig 2B shows that this axon (arrows) from the upper cell turns upward into the vicinity of a dendrite (arrowheads) belonging to the lower cell. There the axon (arrows) appears to spiral around a neurite (a putative dendrite) of the lower cell.

These findings indicate densely packed neurites that originate within MNTB that can reach as far as ~40 μm axial distance. This provides evidence that in order to trace single MNTB neurons’ collaterals it is important to produce only sparse labeling of MNTB cells. Furthermore, our findings confirmed that only those neurons that have been electroporated following dye puff became labeled from the soma to the neurites. While those neurons that got labeled passively from the puff alone never exhibited labeled neurites.

### The morphology of axonal endings and labeled cells

**Fig 3:**
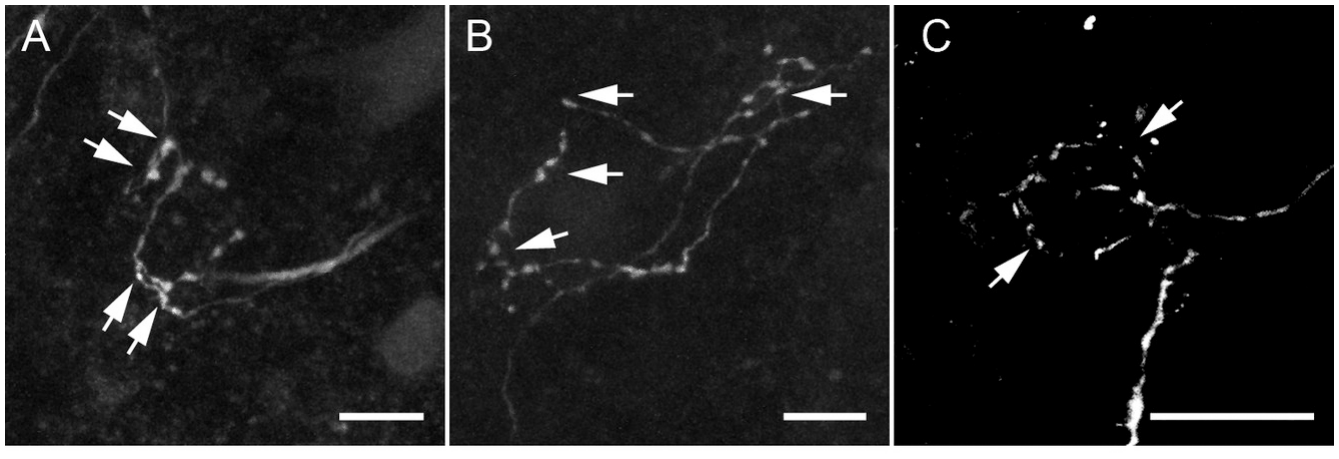
Types of axonal endings formed by neurites in the MNTB. A: Structure with bouton-like swellings formed on the branches which form a circular shape (arrows). B: More classic axonal branches with bouton-like swellings that are thin and spread out (arrows). C: A neuron that formed a terminal (arrows) on its collateral in very close proximity to its originating soma. Scale bars: 10 μm for panels A, B, and 20 μm for panel C.

Biocytin-filled collaterals terminating within the MNTB form various patterns (Fig 3). Generally, we observed that terminals wrapped around putative cell bodies (Fig 3A and 3C) and larger branches split into smaller arborizations (Fig 3A and 3C) before encapsulating the soma (arrows in Fig 3A and 3C). In some cases, neurites appear to wrap around neighboring somata, in close vicinity to the cell body from which they originated (Fig 3C arrows indicate neurites).

Additionally, we also observed axonal endings that have small branches with characteristic swellings, presumably boutons (Fig 3B, arrows) that divide into multiple smaller branches. These terminals also appear to pass near the surface of a cell soma, forming *en passant* terminals, rather than wrapping around the cells.

**Fig 4:**
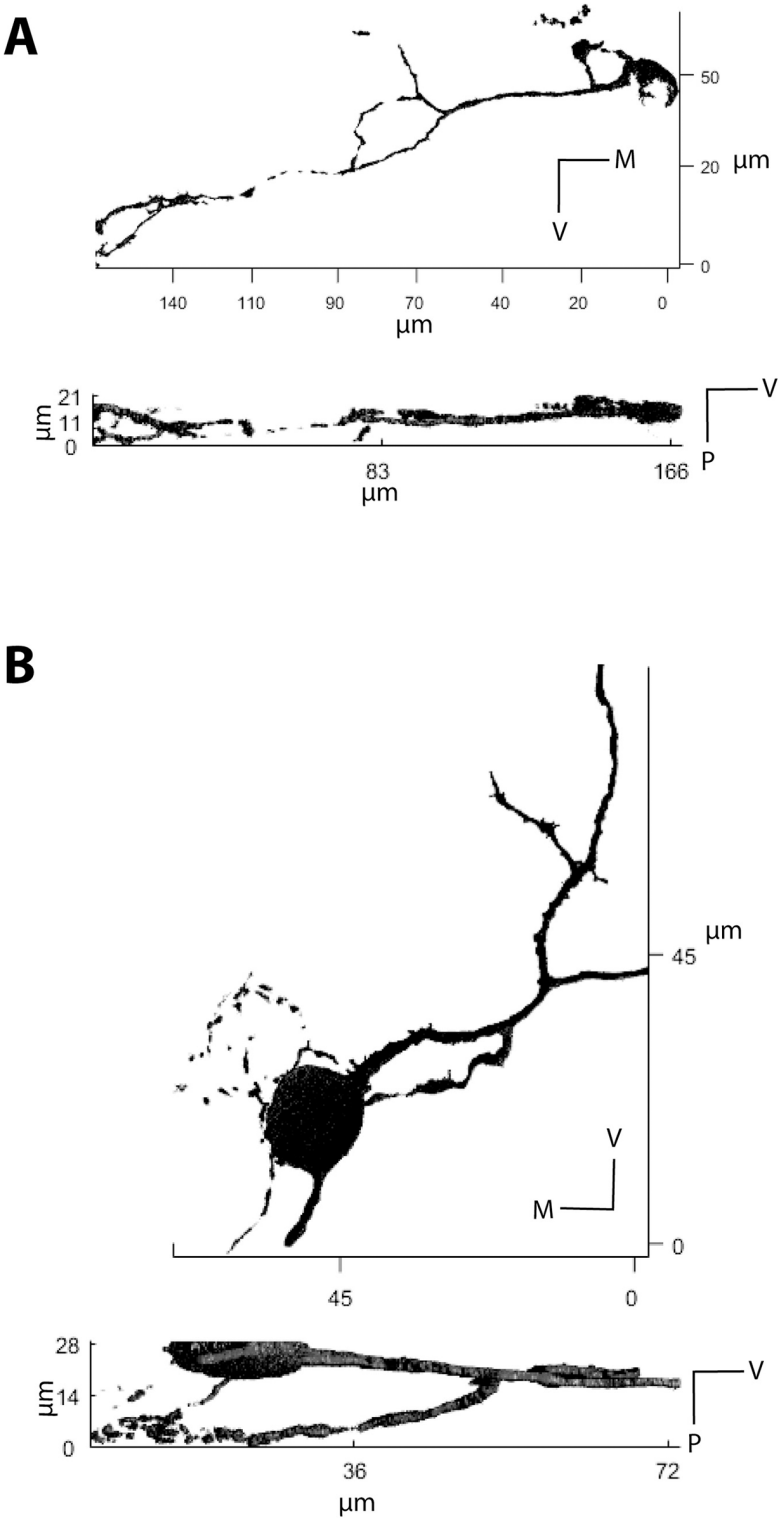
Examples of 3D reconstructions of two biocytin labeled neurons. The reconstructions are based on intensity thresholds and a contiguous pixel cluster algorithm (see Methods), and show that the neurites of the labeled neurons projected dorsally (A), or ventrally (B) but remained within the range of a 14 to 30 μm depth within the coronal plane of the slice (z—axis represents the rostro-caudal direction).

We traced the biocytin filled somata and their neurites, and found that in the 17 cases in which neurites were successfully labeled for longer distances (60 μm to 140 μm), six produced MNTB to MNTB collaterals. Of these, 3D-reconstructions were prepared to analyze the direction of their projections and the depth at which their neurites travel. Neurites predominantly traveled in the medio-lateral direction and remained largely within the coronal plane. Fig 4 shows two examples, in which neurites traveled predominantly in the medio-lateral direction, and remained within 30 μm of the coronal plane (Fig 4; z—axis represents rostro-caudal direction).

In order to further confirm our findings from 3D analysis we conducted a series of parasagittal and horizontal slice preparations and found that labeled neurons indeed projected in the medio-lateral plane rather than rostro-caudal plane (n = 8, data not shown).

### Identity of synapses in the MNTB

In order to further identify the putative axonal terminals, we used pre- and postsynaptic antibodies against SNAP-25 and gephyrin. SNAP-25 is a marker for presynaptic terminals used to identify membrane bound proteins involved in membrane vesicle fusion and exocytosis [35–37]. Gephyrin is a postsynaptic marker, labeling an anchor protein for postsynaptic GABA_A_ and glycine receptors [38–40].

**Fig 5:**
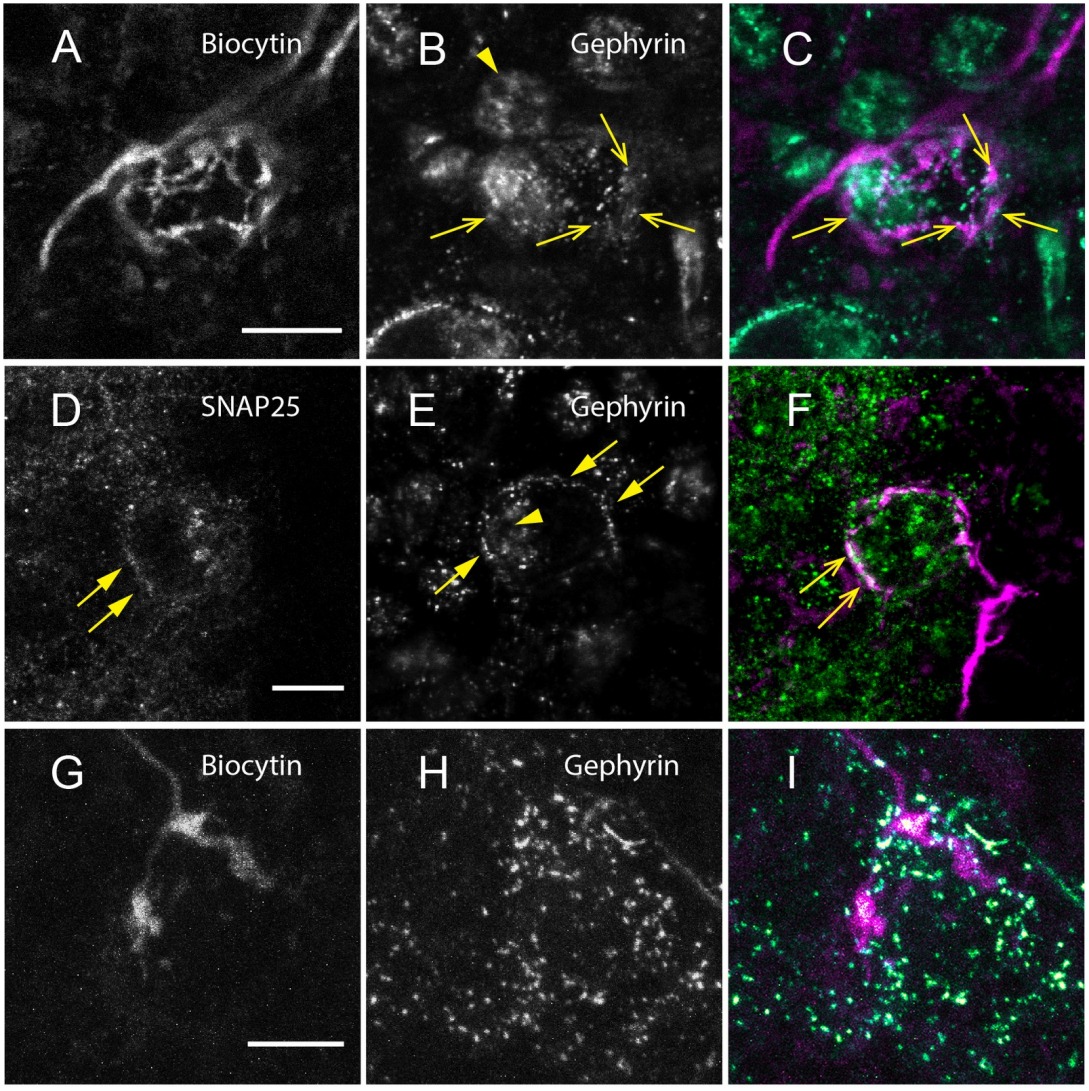
Immunohistochemistry in combination with tracer label suggests that MNTB to MNTB collaterals form functional synapses. A: The compact circular structure labeled in neuron shown in Fig 1B (box). B: Gephyrin staining localized to the cell membrane (arrows) and surrounding extra-nuclear area (arrowheads). C: The overlap of the two channels shown in A and B reveals gephyrin (cyan) juxtaposed to the biocytin labeled axon (magenta, open arrows). D: The presynaptic label SNAP-25 localized at the circumference of the cell soma (not labeled) and E: Gephyrin (arrows and arrowheads as above). F: The overlay of gephyrin (cyan), SNAP-25 (green) and the corresponding axonal ending (magenta) shows very close proximity of neurite with the presynaptic and postsynaptic density (open arrows). G: A third terminal, which appears to form rudimentary fenestrations. H: Gephyrin staining shows a circular shape that suggests a post synaptic cell body. I: The gephyrin label (cyan) seems to be concentrated around the labeled terminal (magenta) that partly encapsulates the putative postsynaptic soma. Scale bars: 10 μm for all panels.

Fig 5A–5C shows the same axonal ending as shown in Fig 1B (box) but with additional anti-gephyrin antibody-labeling. The panels show gephyrin label localized to the membrane of the postsynaptic cell soma, indicating the presence of postsynaptic densities (marked by yellow arrows; Fig 5B). Additional extra-nuclear staining, most likely due to protein translation in the cell soma, is marked by arrowheads (Fig 5B). The overlay of biocytin (magenta) with gephyrin (cyan) is shown in Fig 5C, indicating that the gephyrin clusters align well with the presynaptic axonal elements of the collaterals (Fig 5C, yellow open arrows).

We further confirmed that the postsynaptic structures identified by gephyrin label also coincide with presynaptic SNAP-25 label that was present within the biocytin labeled axons (Fig 5D, 5E and 5F). Fig 5E shows postsynaptic labeling of gephyrin (arrows) aligned well with the cell membrane and non-targeted extra-nuclear staining (arrowheads). Fig 5D shows presynaptic label with SNAP-25 antibody (arrows) present within the structure of the axon (Fig 5F). Fig 5G represents another example of a biocytin labeled neurite that is juxtaposed with a postsynaptic gephyrin (Fig 5H). The terminal coincides spatially with a high concentration of gephyrin antibody-labeling (Fig 5I).

### Frequency of occurrence of MNTB to MNTB collaterals

**Fig 6:**
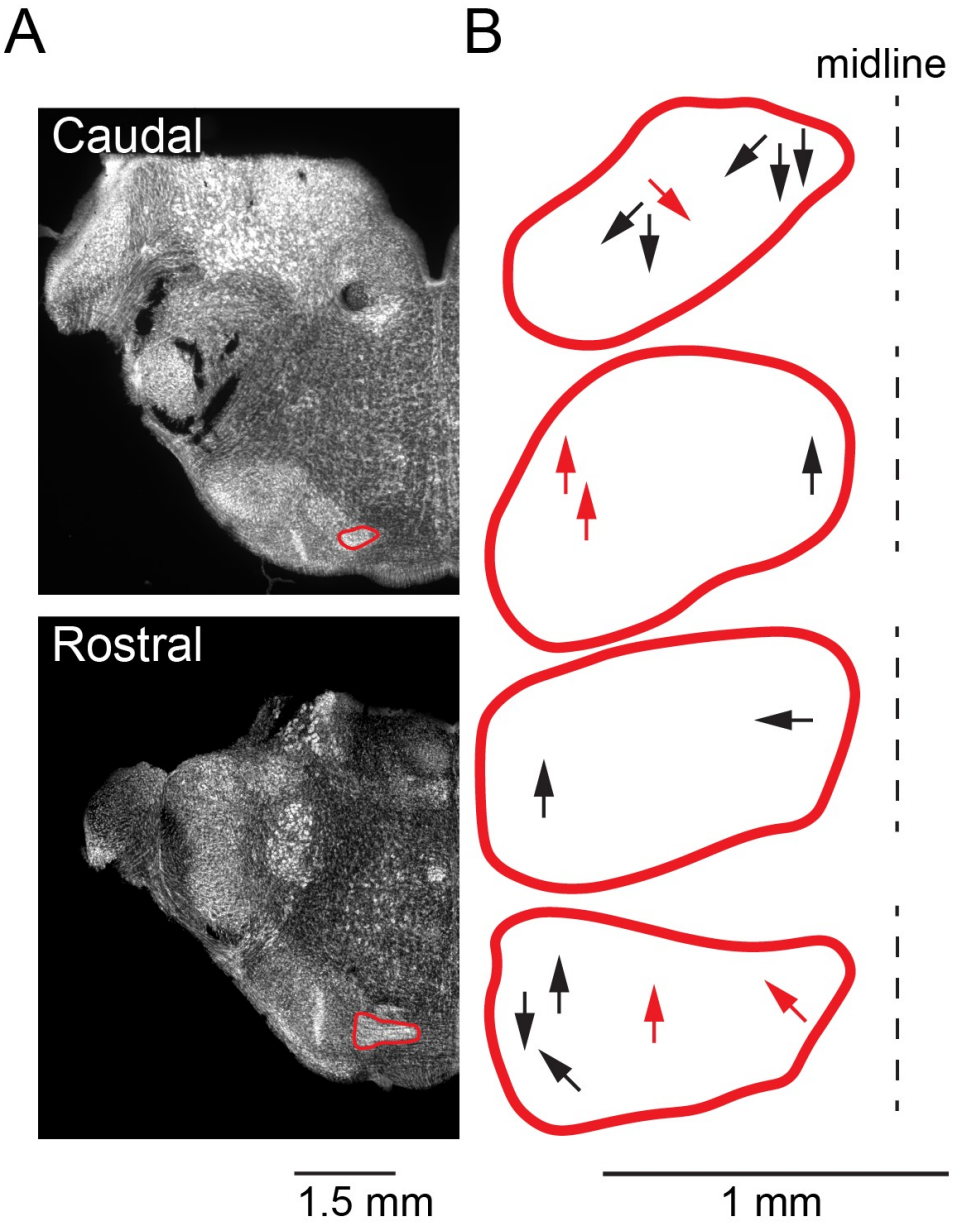
Schematic summary of the location and projection direction of labelled MNTB principal cells. A: The two coronal sections of the gerbil MNTB labeled with Nissl, representing the near to rostral and near to caudal end of the MNTB (rostral at– 4.9 mm, caudal at– 5.5 mm relative to Bregma). B: Location of origin and projection directions of all labeled neurons in which the total neurite length was at least 60 μm, shown in the form of arrows in each rostro-caudal section of the MNTB (red outlines top to bottom represent caudal to rostral). The arrows point in the direction of travel of the longest neurite originating from each labeled neuron, and the arrow origin indicates the approximate location of the labeled neuron in the medio-lateral axis of the MNTB normalized to the midline on the right (dashed line). Red arrows indicate neurons that have axons with returning collaterals within the MNTB. Represented are the 16 cases found in coronal preparations out of the total of 17 cases. One additional case (Fig 5G) found in a horizontal section is not shown here.

We sought to determine the frequency of collaterals of MNTB principal neurons; whether these collaterals are limited to certain areas of MNTB, and if they project to other MNTB neurons in close vicinity of their origin, or more distantly located neurons. To this end, we analyzed the projections of the 17 filled neurites of 60 μm or more. Of these 17 fills, 16 were performed in coronal sections (Fig 6), and one was performed in a horizontal section. For each fill in a coronal section, we recorded the rostro-caudal location of the soma, and the direction of travel of the longest neurite emerging from this filled soma. Fig 6A shows a microphotograph of the most rostral and the most caudal half-section in the coronal plane of a P18 gerbil brainstem. The rostral section corresponds to approximately Bregma -4.9 mm, and the caudal section corresponds to approximately Bregma -5.5 mm (modified for younger animals from the Gerbil Brain Atlas; Loskota, 1974). We traced the location of filled somata and locations of labeled neurites, and mapped both into the sketches of the MNTB (Fig 6B). The sketches top to bottom represent MNTB location in caudal to rostral order. The points of origin of the arrows represent the somata, and direction of the arrows represent the projection direction of the longest neurite emerging from a soma. Red arrows indicate neurons that have axons with returning collaterals within the MNTB (5 neurons). We analyzed travel within the medio-lateral axis in a coronal section of MNTB, and normalized to the location of the midline on the right (dashed line).

Fig 6B shows the data for the 16 neurons that were filled in coronal sections. Not shown is one last case (shown in Fig 5G), found in a horizontal preparation. This neuron was located in the latero-caudal part of the ventral MNTB and was projecting to medio-rostral direction.

In summary, our data suggest that returning collaterals originating from MNTB neurons provide inhibitory input to other MNTB neurons located in the vicinity of the originating cell. Moreover, we used immunohistochemistry to identify and characterize pre- and postsynaptic terminals formed by these biocytin-filled collaterals. Finally, we showed that the terminals consist of pre- and postsynaptic markers, suggesting that these are functional synapses and not merely cases in which a neurite happens to pass close to a MNTB neuron. Although clearly present, the frequency of these collaterals in the MNTB is low, with only about a quarter to a third of MNTB axons forming collaterals to other MNTB neurons.

## Discussion

### Main findings

Our results demonstrate that subpopulations of MNTB neurons receive inhibitory inputs from other MNTB neurons. While the existence of such collaterals had been suggested by various other authors [9,23,41], we demonstrate here directly that these collaterals actually innervate MNTB postsynaptic neurons via functional inhibitory chemical synapses. In our definition, a "functional" synapse consists of an afferent axon ending in a presynaptic terminal and a corresponding postsynaptic density. We also found that only a relatively small portion of MNTB neurons receive this type of collateral inhibition.

Our conclusions are supported by the following results: 1) biocytin labeling of single MNTB neurons revealed recurrent collaterals within the MNTB, 2) a low number of biocytin labeled MNTB neurons per brain section allowed for precise tracing of collaterals and revealed synaptic terminals located close to cell bodies of MNTB neurons, 3) immunohistochemical labeling against the presynaptic marker SNAP-25 together with labeling against the inhibitory postsynaptic marker gephyrin, combined with biocytin-positive neuronal collaterals revealed inhibitory synapses formed by the collaterals, 4) only about a quarter to a third of successfully labeled neurons had either returning collaterals, or formed terminals within the MNTB.

We also found that the returning collaterals form two types of synaptic connections: the collateral either 1) divides into smaller branches with characteristic bouton-like swellings or 2) forms round and compact termini. Furthermore, our data suggest that the collaterals generally synapse on nearby MNTB neurons. We also found no evidence that the synapses follow any tonotopically systematic distribution, although our sample size might be too small to make this determination.

### Frequency of occurrence of MNTB—MNTB collaterals

Our findings that the returning collaterals occur at a low frequency, and that their endings are located on nearby cell bodies in the MNTB are in agreement with previous observations from several species: gerbil and bat, [9], and cat [23,41]. One of these studies comments on the low frequency of these collaterals (between 3–30%, [9]), which is consistent with our findings. Additionally, Guinan and Li [41] observed, using *in-vivo* electrophysiological studies in the cat brainstem, a small subset of neurons with "peculiar spiking behavior", and suggested that this could potentially be explained by a recurrent inhibitory input. Our findings, together with these previous studies, support the existence of returning inhibitory collaterals between principal neurons in the MNTB, although these collaterals seem to be present only between a small subset of MNTB neurons.

The method of injecting individual neurons with biocytin under visual control allows for specific labeling of neurons, eliminates bulk injection which usually generates a very bright area at the injection site, and allows for the injection of morphologically identified single principal neurons that proved to be very useful for finding neuronal collaterals. Labeling of the neurons in 180–300 μm thick slices ensured that the majority of neurites were present within the section. Further re-slicing to 50 μm sections to facilitate antibody penetration might, however, have resulted in some loss of neurites, or cell bodies. Because of this, our estimate of the frequency of these collaterals might underestimate the actual number of collaterals. However, our findings are still in good agreement with the existing, albeit scant literature on this question.

Previous anatomical and physiological studies have shown that virtually every MNTB neuron receives substantial glycinergic inhibition [20,21,24,29] and that the main source of this inhibition is the ipsilateral VNTB [20]. The single cell fillings used here specifically labeled inhibition originating from single MNTB neurons only, thereby isolating these projections from other possible inhibitory inputs to MNTB neurons. Because of the methodology used here, our results are not in contradiction with these other studies that show that most, if not all MNTB principal neurons, receive glycinergic input. Rather, it appears that many if not all MNTB neurons receive inhibitory inputs from the VNTB and in addition, a smaller subset of MNTB neurons receive a second inhibitory amino acid input which originates intrinsically (i.e. from another MNTB neuron).

### Developmental considerations

The innervation of MNTB principal cells by excitatory glutamatergic terminals from VCN begins during the embryonic stage at E17 [42,43]. The VCN tonotopic organization is reached by E15.5 [44]. Therefore, some synaptic activity might already occur at this early stage of MNTB development. The next large wave of changes in MNTB physiology coincides with the calyx formation at around P2 [42,45]. Further development of MNTB cells occurs in stages coinciding with the calyx of Held development, resulting in mature physiological properties by P14 [42,46]. While less is known about the developmental pattern of inhibitory projections to MNTB principal neurons, inhibitory currents evoked at presynaptic calyces appear to be developmentally stable around P14 [47]. MNTB efferents to LSO undergo a long maturational period during which some pruning of terminals established within the LSO is happening for up to three weeks postnatally [48], although the projections from the MNTB to the LSO themselves are established during the neonatal period and remain from that time onward. MNTB efferents to MSO show physiologically mature properties by age P17 [49]. The work discussed here suggests that all projections between auditory brainstem nuclei are established and stable well before hearing onset, although some pruning and maturation of physiological properties still happens during the first week after hearing onset. Importantly, there is no evidence in the literature suggesting that entire projections are eliminated after hearing onset.

The results presented in our study are based on an analysis of brainstem slices from gerbils ranging in age from P13 –P17 (i.e. during the first week after hearing onset (Table 1)), making it highly unlikely that the MNTB to MNTB collaterals described here might be developmentally transient. Nevertheless, we cannot rule out the possibility that the terminals described here may still undergo some minor morphological changes.

### Significance of inhibitory inputs to MNTB neurons

Our data do not address the functional significance of these inhibitory inputs, but we note that virtually all MNTB neurons exhibit a tonic firing pattern when stimulated by sound *in-vivo* [18,23,50–52], even though there is a high probability that they receive at least the extrinsic inhibitory input from VNTB [20]. This suggests that this inhibitory input may not completely suppress MNTB firing but rather interact with the calyceal excitation in a subtler and specific way. A recurrent inhibitory collateral as described here could be activated when the tonically firing principal cell from which it originates fires a series of action potentials for the duration of a presented sound stimulus. These spikes would then travel along the outgoing axon and arrive at the presynaptic terminal of the innervated target cell a moment later. Since these inhibitory collaterals innervate nearby principal cells, presumably with similar best characteristic frequencies, we postulate that the physiological properties of the collaterals described here should include a slightly longer latency than the corresponding calyceal excitation. Also, the inhibition should have tonic properties, since MNTB neurons have tonic firing properties, and should be tuned to frequencies either slightly lower or slightly higher than the principal neuron’s excitatory input is tuned to. The process of lateral inhibition, which would serve to sharpen frequency selectivity for example, matches these criteria. Several authors [9,24,52] have described a form of lateral inhibition that maps well onto the properties described here. However, we are not sure why only a small subset of MNTB neurons should have such a lateral inhibition.

Alternatively, the cells contacted by returning collaterals may actually respond to the same sound frequency (e.g., on Best Frequency inhibition), not just a similar sound frequency. In this case, the physiological role of the collaterals may be to increase temporal fidelity of sound coding, such as to sharpen the onset responses of MNTB neurons to sound stimuli and/or to increase the phase locking of responses to ongoing stimulation [53]. Future *in-vivo* experiments in combination with pharmacological manipulations of inhibition may test this scenario.

### Summary

In summary, we demonstrated that efferent axons from approximately a quarter to a third of MNTB principal neurons form returning inhibitory collaterals on to nearby MNTB principal neurons. Future studies will determine the functional significance of these collaterals, and how this type of inhibitory input interacts with the previously demonstrated inhibitory input from the ipsilateral VNTB.

## Supporting Information

S1 Fig Raw Data: a confocal stack of 54 virtual sections, collected on Olympus FV1000, UPLSAPO obj. 60X W, 1.2N.A. Image resolution 1024x1024, 16 bit, voxel size: x, y, z = 0.207 x 0.207 x 0.4 microns^3^. Biocytin label visualized with Extravidin TRITC. This supporting file can be opened with Fiji (Fiji-win64-20140602), which is a distribution of imageJ (NIH) and includes Bio-Formats plugin (http://imagej.net/Fiji/Downloads).
(ZIP)

S2 Fig Raw Data: a confocal stack of 48 virtual sections, collected on Olympus FV1000, UPLSAPO obj. 60X W, 1.2N.A. Image resolution 1024x1024, 16 bit, voxel size: x, y, z = 0.09 x 0.09 x 0.6 microns^3^. Biocytin label visualized with Extravidin TRITC. This supporting file can be opened with Fiji (Fiji-win64-20140602), which is a distribution of imageJ (NIH) and includes Bio-Formats plugin (http://imagej.net/Fiji/Downloads).
(ZIP)

S3 Fig Raw Data: a confocal stack of 19 virtual sections, collected on Olympus FV1000, UPLSAPO obj. 60X W, 1.2N.A. Image resolution 1024x1024, 16 bit, voxel size: x, y, z = 0.207 x 0.207 x 0.7 microns^3^. Biocytin label visualized with Extravidin TRITC. This supporting file can be opened with Fiji (Fiji-win64-20140602), which is a distribution of imageJ (NIH) and includes Bio-Formats plugin (http://imagej.net/Fiji/Downloads).
(TIF)

S4 Fig Raw Data: a confocal stack of 19 virtual sections, collected on Olympus FV1000, UPLSAPO obj. 60X W, 1.2N.A. Image resolution 1024x1024, 16 bit, voxel size: x, y, z = 0.207 x 0.207 x 0.7 microns^3^. Primary antibody mouse monoclonal antibody against gephyrin from Synaptic Systems, cat# 147011, visualized by a secondary antibody goat anti-mouse conjugated with Alexa Fluor 488, Invitrogen/Molecular Probes cat# A11029. This supporting file can be opened with Fiji (Fiji-win64-20140602), which is a distribution of imageJ (NIH) and includes Bio-Formats plugin (http://imagej.net/Fiji/Downloads).
(TIF)

## References

[1] Thompson AM, Schofield BR. Afferent projections of the superior olivary complex. Microsc Res Tech. 2000;51: 330–54. 10.1002/1097-0029(20001115)51:4<330::AID-JEMT4>3.0.CO;2-X 11071718

[2] Grothe B, Pecka M, McAlpine D. Mechanisms of sound localization in mammals. Physiol Rev. 2010;90: 983–1012. 10.1152/physrev.00026.2009 20664077

[3] Ashida G, Carr CE. Sound localization: Jeffress and beyond. Current Opinion in Neurobiology. 2011 pp. 745–751. 10.1016/j.conb.2011.05.008 21646012 PMC3192259

[4] Borst JGG, Soria van Hoeve J. The Calyx of Held Synapse: From Model Synapse to Auditory Relay. Annu Rev Physiol. 2012;74: 199–224. 10.1146/annurev-physiol-020911-153236 22035348

[5] Morest DK. The collateral system of the medial nucleus of the trapezoid body of the cat, its neuronal architecture and relation to the olivo-cochlear bundle. Brain Res. 1968;9: 288–311. 5679830 10.1016/0006-8993(68)90235-7

[6] Warr WBruce. Fiber degeneration following lesions in the multipolar and globular cell areas in the ventral cochlear nucleus of the cat. Brain Res. 1972;40: 247–270. 10.1016/0006-8993(72)90132-1 5027165

[7] Friauf E, Ostwald J. Divergent projections of physiologically characterized rat ventral cochlear nucleus neurons as shown by intra-axonal injection of horseradish peroxidase. Exp Brain Res. 1988;73: 263–284. 10.1007/BF00248219 3215304

[8] Spirou G a, Brownell WE, Zidanic M. Recordings from cat trapezoid body and HRP labeling of globular bushy cell axons. J Neurophysiol. 1990;63: 1169–90. Available: http://www.ncbi.nlm.nih.gov/pubmed/2358868 2358868 10.1152/jn.1990.63.5.1169

[9] Kuwabara N, DiCaprio R.A, Zook J., Kuwabara N, DiCaprio R a, Zook JM. Afferents to the medial nucleus of the trapezoid body and their collateral projections. J Comp Neurol. 1991;314: 684–706. 10.1002/cne.903140405 1816271

[10] Smith PH, Joris PX, Carney LH, Yin TC. Projections of physiologically characterized globular bushy cell axons from the cochlear nucleus of the cat. J Comp Neurol. 1991;304: 387–407. 10.1002/cne.903040305 2022755

[11] Held H. Die centrale gehörleitung Arch Anat Physiol Anat Abt. 1893;

[12] Rodríguez-Contreras A, de Lange RPJ, Lucassen PJ, Borst JGG. Branching of calyceal afferents during postnatal development in the rat auditory brainstem. J Comp Neurol. 2006;496: 214–28. 10.1002/cne.20918 16538676

[13] HARRISON JM, WARR WB. A study of the cochlear nuclei and ascending auditory pathways of the medulla. J Comp Neurol. 1962;119: 341–79. 13952992 10.1002/cne.901190306

[14] Elverland HH. Ascending and intrinsic projections of the superior olivary complex in the cat. Exp brain Res. 1978;32: 117–34. 658183 10.1007/BF00237396

[15] Spangler KM, Warr WB, Henkel CK. The projections of principal cells of the medial nucleus of the trapezoid body in the cat. J Comp Neurol. 1985;238: 249–262. 10.1002/cne.902380302 4044914

[16] Casey MA, Feldman ML. Aging in the rat medial nucleus of the trapezoid body. II. Electron microscopy. J Comp Neurol. 1985;232: 401–13. 10.1002/cne.902320311 3973099

[17] Tollin DJ. The lateral superior olive: a functional role in sound source localization. Neurosci. 2003;9: 127–143. 10.1177/1073858403252228 12708617

[18] Kopp-Scheinpflug C, Lippe WR, Dörrscheidt GJ, Rübsamen R. The medial nucleus of the trapezoid body in the gerbil is more than a relay: comparison of pre- and postsynaptic activity. J Assoc Res Otolaryngol. 2003;4: 1–23. 10.1007/s10162-002-2010-5 12098017 PMC3202451

[19] Hermann J, Pecka M, von Gersdorff H, Grothe B, Klug A. Synaptic transmission at the calyx of Held under in vivo like activity levels. J Neurophysiol. 2007;98: 807–20. 10.1152/jn.00355.2007 17507501

[20] Albrecht O, Dondzillo A, Mayer F, Thompson JA, Klug A. Inhibitory projections from the ventral nucleus of the trapezoid body to the medial nucleus of the trapezoid body in the mouse. Front Neural Circuits. 2014;8: 83 10.3389/fncir.2014.00083 25120436 PMC4114201

[21] Awatramani GB, Turecek R, Trussell LO. Inhibitory control at a synaptic relay. J Neurosci. 2004;24: 2643–2647. 10.1523/JNEUROSCI.5144-03.2004 15028756 PMC6729505

[22] Green JS, Sanes DH. Early Appearance of Inhibitory Input to the MNTB Supports Binaural Processing During Development. J Neurophysiol. 2005;94: 3826–3835. 10.1152/jn.00601.2005 16120660

[23] Smith PH, Joris PX, Yin TC. Anatomy and physiology of principal cells of the medial nucleus of the trapezoid body (MNTB) of the cat. J Neurophysiol. 1998;79: 3127–3142. 9636113 10.1152/jn.1998.79.6.3127

[24] Kopp-Scheinpflug C, Dehmel S, Tolnai S, Dietz B, Milenkovic I, Rübsamen R. Glycine-mediated changes of onset reliability at a mammalian central synapse. Neuroscience. IBRO; 2008;157: 432–445. 10.1016/j.neuroscience.2008.08.068 18840508

[25] Helfert RH, Bonneau JM, Wenthold RJ, Altschuler RA. GABA and glycine immunoreactivity in the guinea pig superior olivary complex. Brain Res. 1989;501: 269–86. 2819441 10.1016/0006-8993(89)90644-6

[26] Lu T, Rubio ME, Trussell LO. Glycinergic Transmission Shaped by the Corelease of GABA in a Mammalian Auditory Synapse. Neuron. 2008;57: 524–535. 10.1016/j.neuron.2007.12.010 18304482

[27] Awatramani GB, Turecek R, Trussell LO. Staggered development of GABAergic and glycinergic transmission in the MNTB. J Neurophysiol. 2005;93: 819–828. 10.1152/jn.00798.2004 15456797

[28] Banks MI, Smith PH. Intracellular recordings from neurobiotin-labeled cells in brain slices of the rat medial nucleus of the trapezoid body. J Neurosci. 1992;12: 2819–2837. Available: http://www.ncbi.nlm.nih.gov/pubmed/1351938 1351938 10.1523/JNEUROSCI.12-07-02819.1992 PMC6575844

[29] Mayer F, Albrecht O, Dondzillo A, Klug A. Glycinergic inhibition to the medial nucleus of the trapezoid body shows prominent facilitation and can sustain high levels of ongoing activity. J Neurophysiol. 2014;112: 2901–15. Available: http://www.ncbi.nlm.nih.gov/pubmed/25185813 10.1152/jn.00864.2013 25185813 PMC4254873

[30] Pfeiffer F, Simler R, Grenningloh G, Betz H. Monoclonal antibodies and peptide mapping reveal structural similarities between the subunits of the glycine receptor of rat spinal cord. Proc Natl Acad Sci U S A. 1984;81: 7224–7. 6095276 10.1073/pnas.81.22.7224 PMC392111

[31] Schmitt B, Knaus P, Becker CM, Betz H. The Mr 93,000 polypeptide of the postsynaptic glycine receptor complex is a peripheral membrane protein. Biochemistry. 1987;26: 805–11. 3032237 10.1021/bi00377a022

[32] Becker CM, Hoch W, Betz H. Sensitive immunoassay shows selective association of peripheral and integral membrane proteins of the inhibitory glycine receptor complex. J Neurochem. 1989;53: 124–31. 2470857 10.1111/j.1471-4159.1989.tb07303.x

[33] Kirsch J, Betz H. Widespread expression of gephyrin, a putative glycine receptor-tubulin linker protein, in rat brain. Brain Res. 1993;621: 301–10. 8242343 10.1016/0006-8993(93)90120-c

[34] Fischer F, Kneussel M, Tintrup H, Haverkamp S, Rauen T, Betz H, et al Reduced synaptic clustering of GABA and glycine receptors in the retina of the gephyrin null mutant mouse. J Comp Neurol. 2000;427: 634–648. Available: http://www.ncbi.nlm.nih.gov/pubmed/11056469 11056469 10.1002/1096-9861(20001127)427:4<634::aid-cne10>3.0.co;2-x

[35] Oyler GA, Higgins GA, Hart RA, Battenberg E, Billingsley M, Bloom FE, et al The identification of a novel synaptosomal-associated protein, SNAP-25, differentially expressed by neuronal subpopulations. J Cell Biol. 1989;109: 3039–52. 2592413 10.1083/jcb.109.6.3039 PMC2115928

[36] Blasi J, Chapman ER, Link E, Binz T, Yamasaki S, De Camilli P, et al Botulinum neurotoxin A selectively cleaves the synaptic protein SNAP-25. Nature. 1993;365: 160–163. 10.1038/365160a0 8103915

[37] Söllner T, Whiteheart SW, Brunner M, Erdjument-Bromage H, Geromanos S, Tempst P, et al SNAP receptors implicated in vesicle targeting and fusion. Nature. 1993;362: 318–324. 10.1038/362318a0 8455717

[38] Triller a, Cluzeaud F, Pfeiffer F, Betz H, Korn H. Distribution of glycine receptors at central synapses: an immunoelectron microscopy study. J Cell Biol. 1985;101: 683–688. 2991304 10.1083/jcb.101.2.683 PMC2113671

[39] Altschuler RA, Betz H, Parakkal MH, Reeks KA, Wenthold RJ. Identification of glycinergic synapses in the cochlear nucleus through immunocytochemical localization of the postsynaptic receptor. Brain Res. 1986;369: 316–320. Available: http://www.ncbi.nlm.nih.gov/pubmed/3008938 3008938 10.1016/0006-8993(86)90542-1

[40] Baer K, Waldvogel HJ, During MJ, Snell RG, Faull RLM, Rees MI. Association of gephyrin and glycine receptors in the human brainstem and spinal cord: An immunohistochemical analysis. Neuroscience. 2003;122: 773–784. 10.1016/S0306-4522(03)00543-8 14622920

[41] Guinan JJ, Li RY. Signal processing in brainstem auditory neurons which receive giant endings (calyces of Held) in the medial nucleus of the trapezoid body of the cat. Hear Res. 1990;49: 321–34. 2292504 10.1016/0378-5955(90)90111-2

[42] Hoffpauir BK, Kolson DR, Mathers PH, Spirou G a. Maturation of synaptic partners: functional phenotype and synaptic organization tuned in synchrony. J Physiol. 2010;588: 4365–4385. 10.1113/jphysiol.2010.198564 20855433 PMC3008845

[43] Hoffpauir BK, Marrs GS, Mathers PH, Spirou GA. Does the brain connect before the periphery can direct?. A comparison of three sensory systems in mice. Brain Res. Elsevier B.V.; 2009;1277: 115–129. 10.1016/j.brainres.2009.02.050 PMC2700192 19272365

[44] Maklad A, Fritzsch B. Development of vestibular afferent projections into the hindbrain and their central targets. Brain Res Bull. 2003;60: 497–510. 12787869 10.1016/s0361-9230(03)00054-6 PMC3901526

[45] Kandler K, Friauf E. Pre- and postnatal development of efferent connections of the cochlear nucleus in the rat. J Comp Neurol. 1993;328: 161–184. 10.1002/cne.903280202 8423239

[46] Taschenberger H, von Gersdorff H. Fine-tuning an auditory synapse for speed and fidelity: developmental changes in presynaptic waveform, EPSC kinetics, and synaptic plasticity. J Neurosci. 2000;20: 9162–9173. 20/24/9162 [pii] 11124994 10.1523/JNEUROSCI.20-24-09162.2000 PMC6773022

[47] Turecek R, Trussell LO. Reciprocal developmental regulation of presynaptic ionotropic receptors. Proc Natl Acad Sci U S A. 2002;99: 13884–9. 10.1073/pnas.212419699 12370408 PMC129792

[48] Sanes DH, Siverls V. Development and specificity of inhibitory terminal arborizations in the central nervous system. J Neurobiol. 1991;22: 837–54. 10.1002/neu.480220805 1663990

[49] Magnusson AK, Kapfer C, Grothe B, Koch U. Maturation of glycinergic inhibition in the gerbil medial superior olive after hearing onset. J Physiol. 2005;568: 497–512. 10.1113/jphysiol.2005.094763 16096336 PMC1474742

[50] Sommer I, Lingenhöhl K, Friauf E. Principal cells of the rat medial nucleus of the trapezoid body: an intracellular in vivo study of their physiology and morphology. Exp brain Res. 1993;95: 223–39. 8224048 10.1007/BF00229781

[51] Lorteije J a M, Rusu SI, Kushmerick C, Borst JGG. Reliability and precision of the mouse calyx of Held synapse. J Neurosci. 2009;29: 13770–13784. 10.1523/JNEUROSCI.3285-09.2009 19889989 PMC6666705

[52] Koka K, Tollin DJ. Linear coding of complex sound spectra by discharge rate in neurons of the medial nucleus of the trapezoid body (MNTB) and its inputs. Front Neural Circuits. 2014;8: 144 10.3389/fncir.2014.00144 25565971 PMC4267272

[53] Tollin DJ, Yin TCT. Interaural phase and level difference sensitivity in low-frequency neurons in the lateral superior olive. J Neurosci. 2005;25: 10648–57. 10.1523/JNEUROSCI.1609-05.2005 16291937 PMC1449742

