# Supplementary figures and images for: Recurrent Inhibition to the Medial Nucleus of the Trapezoid Body in the Mongolian Gerbil (Meriones Unguiculatus)
Source: PLoS One. 2016 Aug 4;11(8):e0160241. doi: 10.1371/journal.pone.0160241 (PMC4973988; doi:10.1371/journal.pone.0160241)

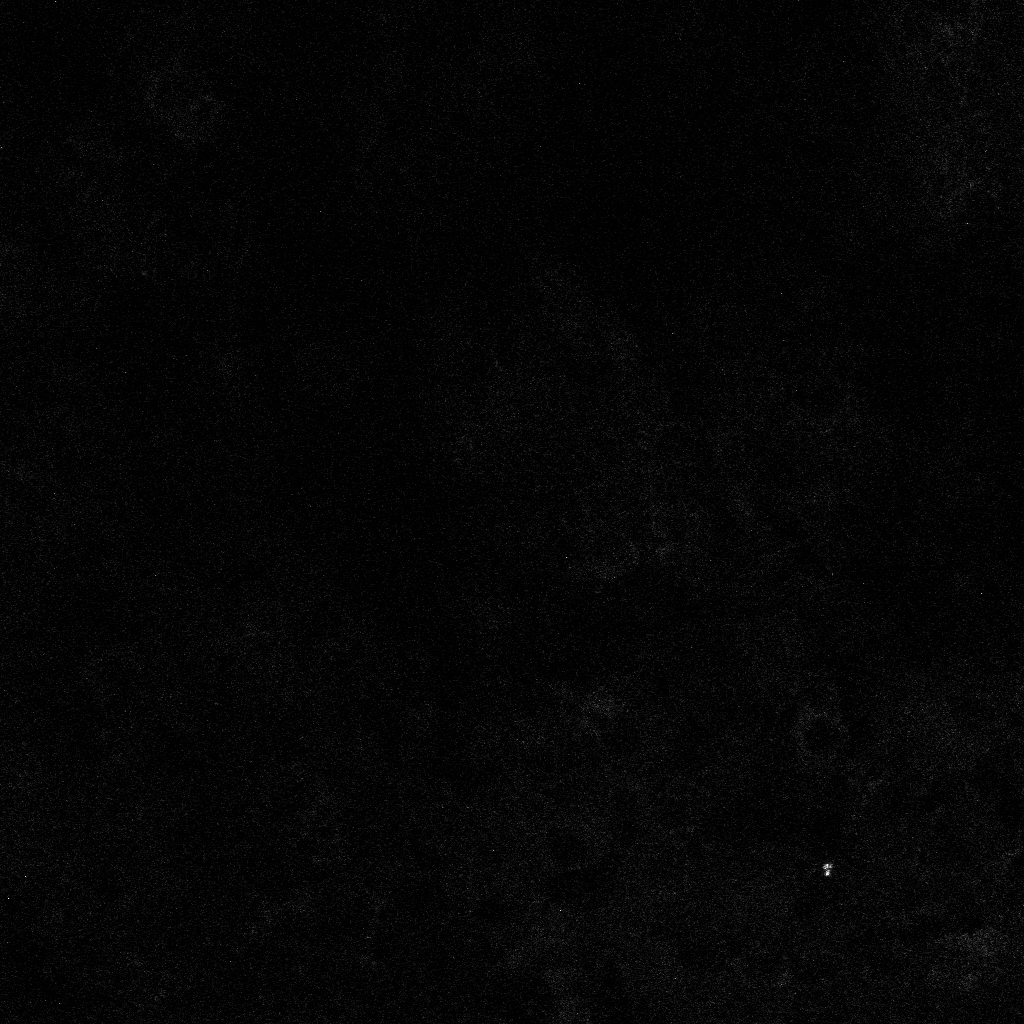

Supplement: S3 Fig — Image resolution 1024x1024, 16 bit, voxel size: x, y, z = 0.207 x 0.207 x 0.7 microns3. Biocytin label visualized with Extravidin TRITC. This supporting file can be opened with Fiji (Fiji-win64-20140602), which is a distribution of imageJ (NIH) and includes Bio-Formats plugin (http://imagej.net/Fiji/Downloads). (TIF) [file pone.0160241.s003.tif]

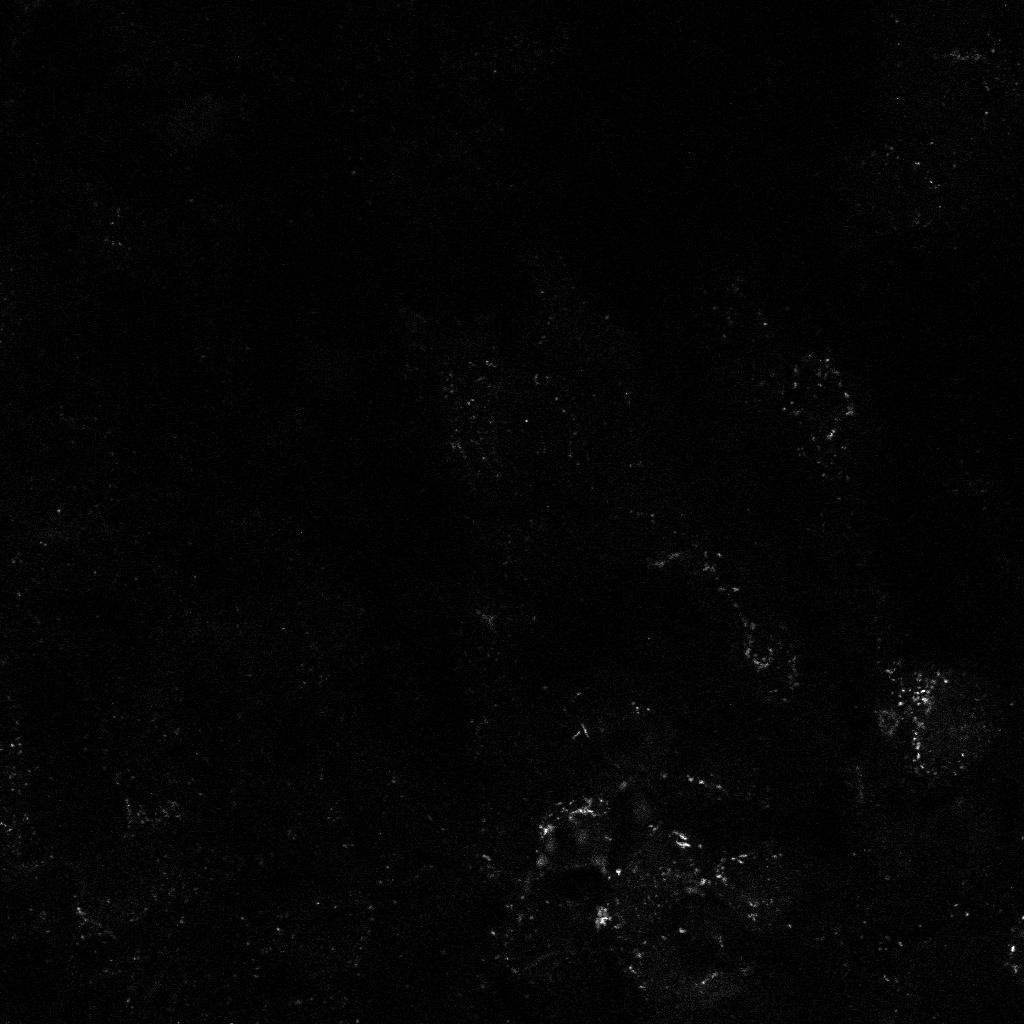

Supplement: S4 Fig — Image resolution 1024x1024, 16 bit, voxel size: x, y, z = 0.207 x 0.207 x 0.7 microns3. Primary antibody mouse monoclonal antibody against gephyrin from Synaptic Systems, cat# 147011, visualized by a secondary antibody goat anti-mouse conjugated with Alexa Fluor 488, Invitrogen/Molecular Probes cat# A11029. This supporting file can be opened with Fiji (Fiji-win64-20140602), which is a distribution of imageJ (NIH) and includes Bio-Formats plugin (http://imagej.net/Fiji/Downloads). (TIF) [file pone.0160241.s004.tif]
